# LDL-lowering therapy and the risk of prostate cancer: a meta-analysis of 6 randomized controlled trials and 36 observational studies

**DOI:** 10.1038/srep24521

**Published:** 2016-04-14

**Authors:** Ping Tan, Shiyou Wei, Zhuang Tang, Liang Gao, Chen Zhang, Pan Nie, Lu Yang, Qiang Wei

**Affiliations:** 1 Department of Urology, Institute of Urology, West China Hospital, Sichuan University, Chengdu, Sichuan, People’s Republic of China; 2 Institute of Urology, West China Hospital, Sichuan University, Chengdu, Sichuan, People’s Republic of China; 3 Department of Cardiovascular and Thoracic Surgery, West China Hospital, Sichuan University, Chengdu, Sichuan, People’s Republic of China; 4 Department of Cardiology, West China Hospital, Sichuan University, Chengdu, Sichuan, People’s Republic of China; 5 Department of Gastrointestinal Surgery, West China Hospital, Sichuan University, Chengdu, Sichuan, People’s Republic of China

## Abstract

The role of statins in preventing prostate cancer is currently a controversial issue. The aim of this review is to investigate the effects of statins use on prostate cancer risk. Electronic databases (the Cochrane Library, PubMed, Medline, Embase, Web of Science, and ClinicalTrials.gov) were searched systematically up to April, 2015. Weighted averages were reported as relative risk (RR) with 95% confidence intervals (CIs). Statistic heterogeneity scores were assessed with the standard Cochran’s Q test and *I^2^* statistic. The pooled estimates of randomized controlled trials (RCTs) and retrospective studies suggest that statins have a neutral effect on total prostate cancer (RR = 1·02, 95% CI: 0·90–1·14; and RR = 0·91, 95% CI: 0·79–1·02, respectively). This research provides no evidence to suggest that the use of statins for cholesterol lowering is beneficial for the prevention of low-grade or localized prostate cancer, although a plausible association between statins use and the reduction risk of advanced (RR = 0·87, 95% CI: 0·82–0·91) or high-grade prostate cancer (RR = 0·83, 95% CI: 0·66–0·99) is observed. Furthermore, it shows that prostate cancer risk does not statistically significant benefit from long-term statins use.

Prostate cancer (PCa) is the most commonly diagnosed cancer among men in the USA^1^. Although the data from the American Society showed that the estimated 5-year survival rate is 98·9%, PCa remains the second most common cause of cancer-related deaths in USA and the leading cause of death in older men^1^. Thus, there is an urgent need for a better understanding of the factors related to the development of PCa and its prognosis.

Statins (3-hydroxy-3-methylglutaryl coenzyme A reductase inhibitors) are the most widely used drugs for lowering cholesterol. Over the past 25 years, there has been increasingly great interest in the antitumour effects of statins, and laboratory research suggests that statins show an inhibitory potential on the growth of PCa, both *in vitro* and *in vivo*^2^,^3^,^4^,^5^. However, clinical studies have not yet shown a consensus as to whether statin use is associated with a decreased (or increased) risk of overall PCa.

Recently, two meta-analyses^6^,^7^ discussed the association of statins with PCa risk; however, they reached contradictory conclusions. However, because of considerable evidence implies that statin use may reduce the risk of PCa, it is both important and necessary to gain a better understanding of whether such therapy can influence disease outcomes. Therefore, we conducted a comprehensive review of all relevant published studies and provided a quantitative assessment of these issues by analysing factors causing inconsistent results.

## Methods

### Study selection

A literature search was performed without language restrictions using the databases of PubMed (Jan 1967–April 2015), MEDLINE (Jan 1967–April 2015), EMBASE (Jan 1990–April 2015), The Cochrane Library, Web of Science, and ClinicalTrials.gov. In addition, a manual search in published articles was conducted to identify additional relevant studies. After removing duplicate publications, two reviewers (Tan & Wei) independently assessed all remaining results by checking titles and abstracts. Studies investigating the association between statins and PCa were considered for further full-text assessment. All randomized controlled trials (RCTs), cohort studies, and case-control studies with both full-text articles and abstracts associated with the topic were considered to be eligible. Letters to the editor, comments, editorials, case reports, and animal studies were excluded. When studies reported outcomes from similar or overlapping databases or cohorts, only data from the most recent publication were included. We adapted a PRISMA (preferred reporting items for systematic reviews and meta-analyses) flow-chart to depict the study selection.

### Data extraction

Data from each study were independently extracted by two reviewers (Tan & Wei) using a standardized data-extraction form. Any disagreements were resolved by consensus or by consultation with a third reviewer (Yang). The following information was checked for each article: first author’s last name, year of publication, location of study, study period, type of study design, mean follow-up time, drugs studied, duration of statin use, study population, number of male subjects, mean age of population, number of total cases of PCa, advanced (defined by the stage of the disease as ‘regional’ or ‘distant’ or the TNM stage within T3-4, N1-3 and M1) and localized PCa cases (defined by the stage of the disease as ‘localized’ or the TNM stage as T1-2, N_0/x_ and M_0/x_.), high (Gleason sum ≥ 7) and low grade PCa cases (Gleason sum <7), PCa cases occurring during short- and long-term statins use (‘long-term’ was defined as ≥5 years of use; ‘short-term’ was defined as <5 years of use), risk estimates [including relative risk (RR), odds ratio (OR) and hazard ratio (HR)] adjusted for the maximum number of confounding variables with corresponding 95% confidence intervals (CIs). In addition, we also tried to contact authors via e-mail to obtain further information that had not been reported in their published articles.

### Quality assessment

Two reviewers (Tan & Wei) independently used the Newcastle–Ottawa Scale (NOS) to assess the quality of the observational studies included (cohort and case-control studies). NOS comprises three parts (selection, comparability, and exposure for case-control studies or outcome for cohort studies) and scores of 4, 2 and 3 are assigned for these three parts, respectively. Studies with scores of 0–3, 4–6 and 7–9 were considered as low, moderate and high quality, respectively. The quality assessment of RCTs was conducted using the modified Jadad scale, which gives the following scores: generation of the allocation sequence (2), concealment of allocation (2), blinding (2), and incomplete outcome data (1). Scores of 1–3 indicate low quality and 4–7 indicate high quality.

### Statistical analysis

RRs and their 95% CIs were used to assess the strength of association between statin use and the risk of PCa in RCTs and retrospective studies. Because HR was broadly equivalent to RR^8^,^9^, HRs were directly considered to be RRs. ORs were converted into RRs using the following formula: RR = OR/[(1 − P_0_) + (P_0_ × OR)], where P_0_ stands for the incidence of PCa in the non-statin use group^10^. We identified heterogeneity between studies using the standard Cochran’s Q test with a significance level of α = 0·10. We also examined heterogeneity with the *I^2^* statistic, which quantifies inconsistency across studies to assess the impact of meta-analysis heterogeneity. An *I^2^* statistic of 50% or more indicates a considerable level of heterogeneity. When heterogeneity was found, we attempted to determine potential sources of heterogeneity by examining individual study and subgroup characteristics. Fixed-effects models were used to pool risk estimates when heterogeneity among studies was considered statistically insignificant. Otherwise, random-effects model was applied to combine the results. We conducted subgroup analyses according to sample size, duration of statin use and stage or grade of PCa. Publication bias was detected using the Egger’s tests. Statistical significance was determined using the two-tailed test, where *P *< 0·05 was considered significant. STATA version 10 (Stata corporation, college station, TX) was employed to conduct all statistical analyses.

## Results

**Figure 1:**
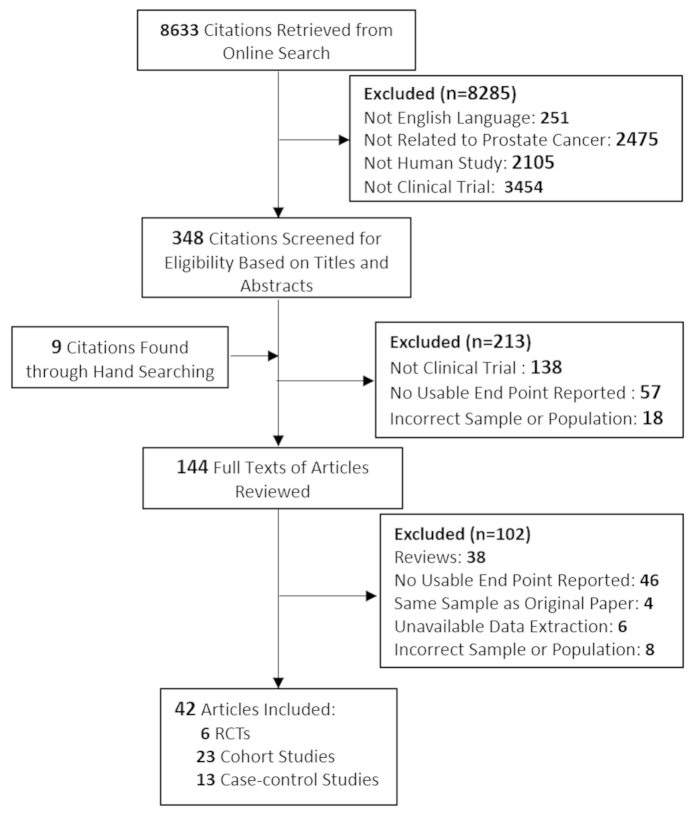
Trial Identification, Inclusion, and Exclusion.

A total of 8,633 articles were identified during the initial search (Fig. 1), and after employing exclusion criteria, a total of 42 studies were included, consisting of 23 cohort studies^11^,^12^,^13^,^14^,^15^,^16^,^17^,^18^,^19^,^20^,^21^,^22^,^23^,^24^,^25^,^26^,^27^,^28^,^29^,^30^,^31^,^32^,^33^, 13 case-control studies^34^,^35^,^36^,^37^,^38^,^39^,^40^,^41^,^42^,^43^,^44^,^45^,^46^ and 6 RCTs^47^,^48^,^49^,^50^,^51^,^52^, all of which involved more than 159,000 PCa cases. The characteristics of the cohort and case-control studies are presented in Supplementary Tables 1 and 2, respectively. Information regarding statins use and the diagnosis of PCa were mainly obtained from medical records and databases, the other sources were self-reported data. The 95% CI of 24 studies included 1·00, showed that no effect had been identified; 11 studies found a significant risk in the reduction of overall PCa in statin users; conversely, seven studies suggested an increased risk.

### Statins and risk of total PCa

**Figure 2:**
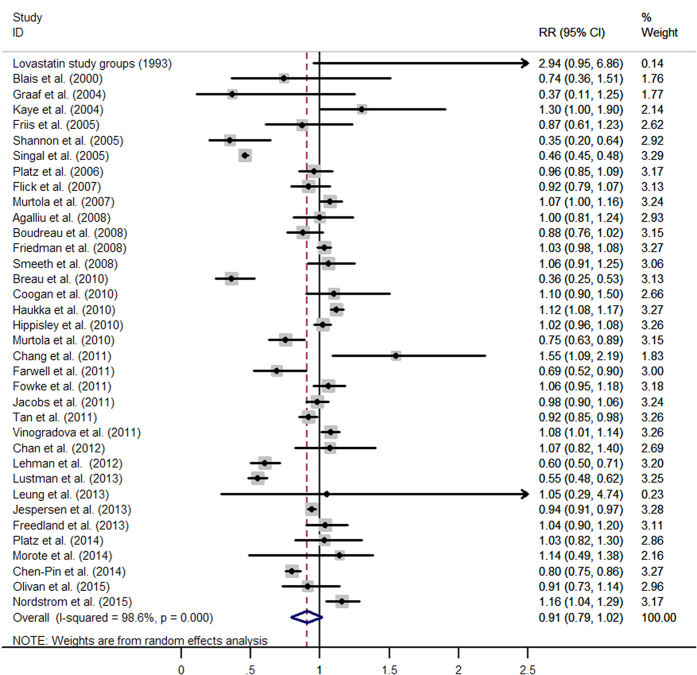
Statins use and risk of total prostate cancer in observational studies. (From random-effects model, RR, relative risk; 95%CI, 95% confidence intervals).

**Figure 3:**
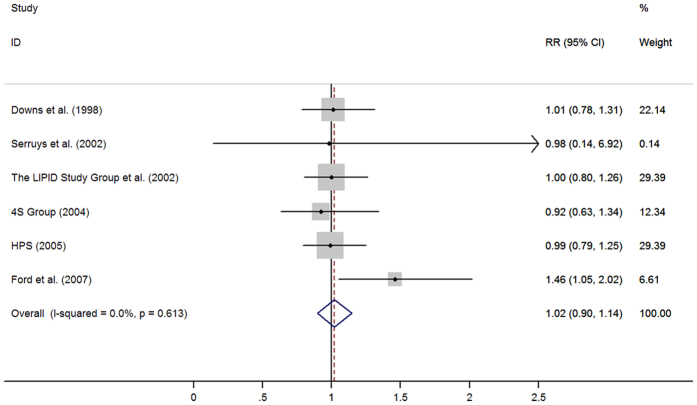
Statins use and risk of total prostate cancer in 6 randomized controlled trials (From Fixed-effects model, RR, relative risk; 95%CI, 95% confidence intervals).

The pooled results from 36 retrospective studies (RR = 0·91, 95% CI: 0·79–1·02) (Fig. 2) and six RCTs (RR = 1·02, 95% CI: 0·90–1·14; I^2^ = 0·0%, *p* = 0·613) (Fig. 3) both suggested that statins have a neutral effect on total PCa. However, results of 23 cohort studies showed an inverse association (RR = 0·90, 95% CI: 0·82–0·99). Cumulative meta-analysis found there was no association between statins use and PCa risk since first studies in 1993 and remained stable after that, only a benefit was noted when Lustman *et al.*^29^ added in 2013. A summary of analyses results is shown in Table 1.

In sensitivity analyses in which one study at a time was excluded and the rest were analysed, the results remained stable and no evident variability was found (data not shown).

### Statins and risk of advanced and localized PCa

**Figure 4:**
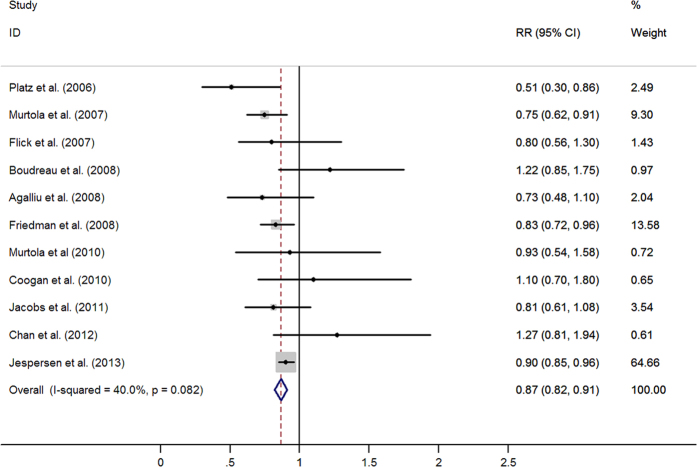
Statins use and risk of advanced prostate cancer (RR, relative risk; 95%CI, 95% confidence intervals).

Eleven studies evaluated exposure to statins and the incidence of advanced PCa. The pooled estimates showed a statistically significant inverse association between statins use and the risk of advanced PCa (RR = 0·87, 95% CI: 0·82–0·91). No significant heterogeneity was observed (I^2^ = 40.0%, *p* = 0·082) (Fig. 4).

Eight studies were available to evaluate the relationship between statins use and the incidence of localized PCa. However, the combined results showed that the association was neutral (RR = 0·98, 95% CI: 0·91–1·06; I^2^ = 71.6%, *p* = 0·001). (see Figure S1).

### High-grade and low-grade PCa

Unexpectedly, the combined results of 15 retrospective studies found a significant association between statins use and the risk of high-grade PCa (RR = 0·83, 95% CI: 0·66–0·99; I^2^ = 90.3%, *p *< 0·001) (Figure S2). While this benefit was null among ten studies for the low-grade PCa (RR = 0·95, 95% CI: 0·88–1·02; I^2^ = 34·0%, *p* = 0·135) (Figure S3).

### Long-term and short-term statin use

**Figure 5:**
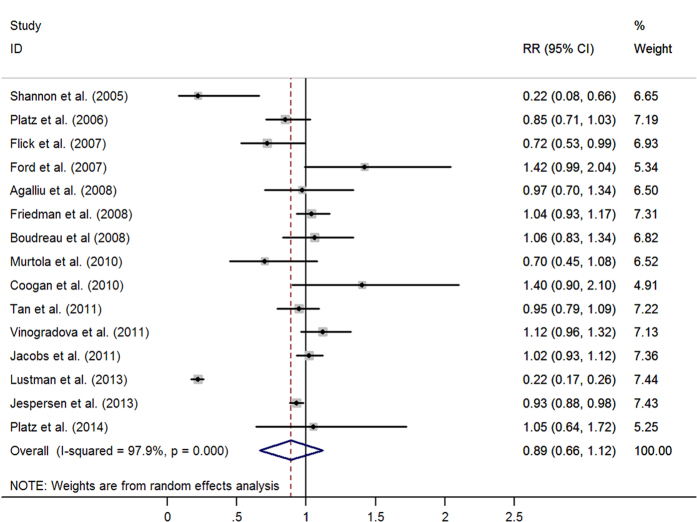
Long-term statins use and risk of total prostate cancer (RR, relative risk; 95%CI, 95% confidence intervals).

The combined RR of 15 trials suggested no statistically significant benefit from the use of long-term statins in relation to the risk of overall PCa (RR = 0·89, 95% CI: 0·66–1·12) (Fig. 5). In relation to long-term statin use, the pooled results showed an association with a decreased risk of advanced PCa (RR = 0·87, 95% CI: 0·79–0·95) and high-grade PCa (RR = 0·79, 95% CI: 0·65–0·92), but no association was observed with localized PCa and low-grade PCa. Synthesis of the available reports that had specifically examined statins use for more than 10 years in relation to total PCa (n = 3) indicated a protective association (RR = 0·92, 95% CI: 0·84–1·00) (Table 2).

Intriguingly, a benefit was noted among short-term statins users (n = 14) (RR = 0·88, 95% CI: 0·78–0·98) (Figure S4). In subgroup analyses, a statistically significant inverse association was identified with advanced PCa, but not with localized PCa.

### Publication bias

**Figure 6:**
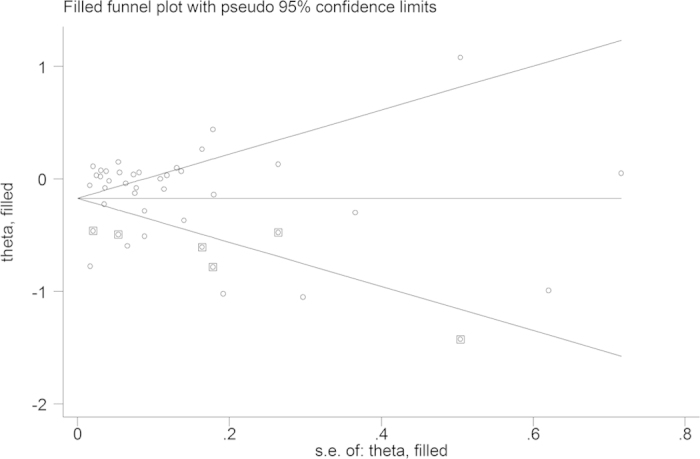
Funnel plot for publication bias.

A potential publication bias was observed (Begg’s test, *p* = 0·002; Egger’s test, *p* = 0·380). Therefore, we performed a sensitivity analysis using the trim and fill method (Fig. 6.). The Filled estimate showed a reverse association (RR = 0·825, 95% CI: 0·737–0·924), means a possibly potential publication bias might exist.

## Discussion

There is no evidence provided by this research to suggest that the use of statins at low doses for managing hypercholesterolemia is beneficial for the prevention of total, low-grade, or localized PCa. This is generally consistent with a previous meta-analysis^6^ included 13 observational studies and six RCTs. Meanwhile, in three other meta-analyses of randomized controlled trials, they also found statins had a neutral effect on cancer and cancer death risk, and no type of cancer was affected by statins use^53^,^54^,^55^. However, a recent meta-analysis^7^ by Bansal *et al.* in 2012 included 27 studies and found approximately 7% reduction risk of total PCa in statins users compared with non-users. This inconsistency is likely to be associated with the inclusion of 16 new studies published after 2011, which suggested that statins lowered the incidence of total PCa. As expected, no association was found between the long-term statins use and the incidence of total PCa in their study. While the results of Bonovas *et al.*^6^ and Bansal *et al.*^7^ both showed an inverse association between statins use and the risk of advanced PCa, which was consistent with the result of our trial. In addition, we found that a benefit was noted in high-grade PCa, to our knowledge, which was found for the first time. While, this result should be approached with caution, as there was significant heterogeneity and upper CI was very close to 1.00.

As described above, the use of statins is both significantly inversely related to the risk of clinically advanced PCa and high-grade PCa. Given the known effects of statins on the PCa cell cycle and apoptosis, especially its ability to decrease the development of existing cancer rather than initiation of cancer, this finding may be plausibly explained. While, this finding may also be explained by a detection bias^32^. Because of the difference in social and economic statuses between statins users and non-users, patients who use statins may have better access to health care and receive greater preventive care, such as PSA screening or prostate biopsies, contributing to the early detection of PCa, thus lowering the risk of advanced/high-grade PCa. Another possible source of detection bias is the influence of statins on serum PSA. Hamilton *et al.*^56^ found that statins users have lower PSA than non-users and that levels of PSA decline after commencing statins use. However, Mondul *et al.*^57^ found that detection bias was unlikely to explain this potential inverse association. Hence, we cannot definitively declare that the observed association between statins and advanced PCa/high-grade PCa is causal or that it should be attributed to varying uptakes of PSA testing between statins users and non-users.

Intriguingly, we found no statistically significant benefit from the long-term use of statins, but a benefit was noted from short-term statins use. Whether this finding is attributed to either residual confounding or type I error of studies is unknown. One possible explanation is various definitions of duration of exposure in each trial and the irregular use of statins in many participants, with months of non-use between periods of use. Hence, the cumulative amount of statin defined daily doses (DDDs) could be small despite the long duration use. It should be noted that the inverse association between the risk of PCa and statins use was dose-dependent with a cumulative amount of statins use^21^. Thus, future studies should take fully into account of influence of cumulative amount of DDDs on the overall statins exposure.

This study has several limitations. First, the combined estimates in this study are inconsistent between cohort and case-control studies in some subgroups. These inconsistencies are likely to be attributed to inherent limitations, notably bias and unmeasured confounding factors existed in observational studies. At this stage, more RCTs would be required to evaluate these relationships. Second, significant heterogeneities were observed in some analyses that we conducted. Fortunately, the heterogeneities lowered down in planned subgroups, reflecting that stage or grade of PCa and period of statins use all contributed to heterogeneities. Furthermore, the number and content of adjusted confounders were varied among studies. Provided it is known that 5α-reductase inhibitors, aspirin and antidiabetic can affect the risk of PCa, which could have produced inaccuracy in the effect estimates. However, these information was unavailable in several studies^18^,^25^,^27^,^29^. To minimize these confounding biases, multivariable adjusted-effect estimates were selected. At last, a potential publication bias was noted among 42 studies, which might be attributed to the lower quality of some literature and the data of some meeting abstracts were unavailable. Thus, the part of our results should be explained with caution.

## Conclusions

Statins have a neutral effect on PCa risk. However, a plausible link was found between a decreased risk of advanced PCa/high-grade PCa and statins use. It is considered that further studies are required to address the risk of overall PCa and clinically important advanced PCa/high-grade PCa among statin users with potential sources that may cause detection bias being well controlled.

## Additional Information

**How to cite this article**: Tan, P. *et al.* LDL-lowering therapy and the risk of prostate cancer: a meta-analysis of 6 randomized controlled trials and 36 observational studies. *Sci. Rep.* **6**, 24521; doi: 10.1038/srep24521 (2016).

## Supplementary Material

Supplementary Information

## Figures and Tables

**Table 1:** The pooled estimates of meta-analysis in subgroups.

			*Pooled estimates*		
*Outcome*		*No. of studies*	RR	95%CI	*I*^*2*^ statistic *P*-Value
*Total PCa*	All studies	42	0.92	0.82 to 1.03	<0.001
	RCTs	6	1.02	0.90 to 1.14	0.613
	Without RCTs	36	0.91	0.79 to 1.02	<0.001
	Cohort studies	23	0.90	0.82 to 0.99	<0.001
	More than 10,000^$^	14	0.91	0.82 to 1.01	<0.001
	Case control studies	13	0.90	0.68 to 1.12	<0.001
	More than 10,000	5	0.85	0.55 to 1.32	<0.001
*Advanced PCa*	All studies	11	0.87	0.82 to 0.91	0.082
	Cohort studies	7	0.82	0.73 to 0.91	0.109
	More than 10,000	6	0.81	0.72 to 0.90	0.161
	Case control studies	4	0.88	0.83 to 0.93	0.164
	More than 10,000	2	0.84	0.71 to 1.00	0.076
*Localized PCa*	All studies	8	0.98	0.91 to 1.06	0.001
	Cohort studies	5	0.95	0.83 to 1.08	<0.001
	More than 10,000	4	0.95	0.83 to 1.08	<0.001
	Case control studies	3	1.00	0.95 to 1.04	0.392
*High-grade PCa*	All studies	15	0.83	0.66 to 0.99	<0.001
	Cohort studies	12	0.84	0.68 to 1.01	<0.001
	More than 10,000	6	0.83	0.57 to 1.08	<0.001
	Case control studies	3	0.79	0.13 to 1.45	<0.001
*Low-grade PCa*	All studies	10	0.95	0.88 to 1.02	0.135
	Cohort studies	7	0.96	0.85 to 1.07	0.091
	More than 10,000	4	0.93	0.79 to 1.11	0.026
	Case control studies	3	0.92	0.75 to 1.10	0.261

Abbreviations:PCa, prostate cancer; 95%CI, 95% confidence intervals; RR, relative risk.

^$^Subgroups analyses in studies included more than 10,000 participants.

**Table 2:** The analysis of relationship between the period of statins use and PCa risk.

		*No. of studies*	*Pooled estimates*		*I*^*2*^ statistic *P*-Value
Outcome			RR	95%CI	
*Statins use less than 5 years*	All studies	14	0.88	0.78 to 0.98	<0.001
*Total PCa*	Cohort studies	9	0.88	0.74 to 1.02	<0.001
	More than 10,000	7	0.85	0.71 to 1.00	<0.001
	Case control studies	5	0.88	0.70 to 1.06	<0.001
	More than 10,000	2	0.98	0.94 to 1.02	0.164
*High-grade PCa*	Cohort studies	4	0.77	0.58 to 0.96	0.125
*Low-grade PCa*	Cohort studies	2	0.92	0.50 to 1.35	0.003
*Advanced PCa*	All studies	9	0.86	0.81 to 0.91	0.670
	Cohort studies	6	0.81	0.71 to 0.91	0.755
	Case-control studies	3	0.88	0.82 to 0.95	0.447
*Localized PCa*	All studies	7	1.02	0.95 to 1.09	0.051
Cohort studies	4	1.00	0.87 to 1.12	0.007	
Case-control studies	3	1.02	0.96 to 1.08	0.996	
*Statins use more than 5 years*	All studies	15	0.89	0.66 to 1.12	<0.001
*Total PCa*	Cohort studies	9	0.84	0.52 to 1.16	<0.001
	More than 10,000	7	0.73	0.50 to 1.06	<0.001
	Case control studies	5	0.89	0.63 to 1.15	<0.001
	More than 10,000	2	1.01	0.84 to 1.20	0.03
*High-grade PCa*	Cohort studies	5	0.79	0.65 to 0.92	0.669
*Low-grade PCa*	Cohort studies	3	0.94	0.71 to 1.16	0.072
*Advanced PCa*	All studies	9	0.87	0.79 to 0.95	0.049
	Cohort studies	6	0.67	0.51 to 0.83	0.164
	Case-control studies	3	0.93	0.84 to 1.02	0.909
	All studies	7	0.97	0.88 to 1.05	0.059
*Localized PCa*	Cohort studies	4	0.94	0.79 to 1.10	0.010
	Case-control studies	3	0.96	0.88 to 1.03	0.859
*Statins use more than 10 years*	Case-control studies	3	0.92	0.84 to 1.00	0.41

Abbreviations: PCa, prostate cancer; 95%CI, 95% confidence intervals; RR, relative risk.

$Subgroups analyses in studies included more than 10,000 participants.
