# Supplementary material for: LDL-lowering therapy and the risk of prostate cancer: a meta-analysis of 6 randomized controlled trials and 36 observational studies
Source: Sci Rep. 2016 Apr 14;6:24521. doi: 10.1038/srep24521 (PMC4830970; doi:10.1038/srep24521)

Supplementary Files:

LDL-lowering therapy and the risk of prostate cancer: a meta-analysis of 6 randomized controlled trials and 36 observational studies

Ping Tan, MD [a, b], Shiyou Wei, MD [c], Zhuang Tang, MD [a, b], Liang Gao, MD [a, b], Chen Zhang, MD [d], Pan Nie, MD [e], Lu Yang, PhD [a, b, *], Qiang Wei, PhD [a, b, *]

Files Legends

TABLE S1. Characteristics of 23 Cohort Studies and 6 RCTs Included in the Meta-analysis.

TABLE S2. Characteristics of 13 Case-control Studies Included in the Meta-analysis.

Figure S1. Statins use and risk of localized prostate cancer.

Figure S2. Statins use and risk of high-grade prostate cancer.

Figure S3. Statins use and risk of low-grade prostate cancer.

Figure S4. Short-term statins use and risk of total prostate cancer.

TABLE S1. Characteristics of 23 Cohort Studies and 6 RCTs Included in the Meta-analysis.

Author, year(country)	Study period(y)	PCa* cases(N)/ Statin group(N)	PCa cases(N)/ Control group(N)	Mean follow-up	Description of exposure	Information source	Quality score
Nordstrom et al. 2015(Sweden)	6(2007-2012)	2321/4825	5233/11923	NR[$]	e	B	8
Platz et al.2014(U.S.)	9(1994-2003)	95/2249	422/7208	>7y	a	A	6
Morote et al.2014(Mediterranean)	6(2006-2011）	232/744	616/1664	NR	a	A	6
Chen-Pin et al. 2014 (U.S.)	10(2003-2013)	3756/71999[1]		6.4y	c	C	8
Lustman et al.2013(Israel)	9(2001-2009)	399/37645	990/29096	NR	d	B	8
Freedland et al. 2013 (U.S.)	NR	1517/6729[2]		4y	a	A	5
Lehman et al. 2012 (U.S.)	6(1999-2005)	152/2937	208/2105	5.2y	c	C	7
Chan et al. 2012 (U.S.)	8(2000-2008)	116/1377	240/3692	7y	c	A	6
Tan et al.2011(U.S.)	10(2000-2010)	565/1022	1842/3182	NR	e	B	6
Jacobs et al.2011(U.S.)	10(1997-2007)	824/7169	3089/45343	NR	g	A	7
Fowke et al. 2011(U.S.)	8(2002-2010)	319/783	525/1365	NR	c	A	7
Farwell et al. 2011(England)	11(1997-2007)	359/41078	187/14797	5.6y	f	B	8
Murtola et al.2010(Finland)	9(1996-2004)	268/6692	1326/16516	6.92y	a	C	8
Hippisley et al. 2010(England & Wales)	6(2002-2008)	7129/990495[2]		NR	b	B	7
Haukka et al. 2010(Finland)	10(1996-2005)	562/5871	489/5057	3.06y	b	C	8
Breau et al. 2010(U.S.)	18(1990-2007)	38/634	186/1813	15.7y	b	A	7
Smeeth et al.2008(U.K.)	12(1995-2006)	312/65517	3213/299158	4.4y	b	B	8
Friedman et al. 2008(U.S.)	9(1994-2003)	1706/192598	NR/1904876	4.91y	d	B	7
Boudreau et al.2008(U.S.)	15(1990-2005)	246/12013	2286/71359	3.3y	e	C	7
Flick et al. 2007(U.S.)	15(1991-2005)	270/22903	618/46144	8.8y	e	B	7
Platz et al. 2006(U.S.)	12(1990-2002)	322/334702[#]	2257/42237[#]	NR	a	A	7
Friis et al. 2005 (Denmark)	13 (1989-2002)	34/6935	1373/160491	3.3y	i	C	6
Lovastatin study groups 1993 (U.S., Canada & Finland)	NR	5/504[4]		4.8y	j	A	7
Ford et al.2007(Scotland)	15(1989-2004)	89/3291	59/3286	13.2y	h	B	7
HPSC. 2005(U.K.)	NR	145/7727	145/7727	5y	k	B	5
4S Group 2004 (Multicenter)	NR	51/1814	55/1803	10y	k	B	7
The LIPID Study Group. 2002(Multicenter)	2(1990-1992)	148/3756	145/3742	8.0y	h	B	4
Serruys et al.2002(Multicenter)	5(1996-2001)	2/844	2/833	3.9y	l	B	5
Downs et al.1998(U.S.)(AFCAPS)	7(1990-1997)	109/2805	108/2803	5.2y	m	B	5

* PCa, prostate cancer; $ NR, not reported; # Person-years at risk. A total of 34989 males in statin and control group.

1, a total of 3756 patients diagnosed with PCa in 71999 males during study period; 2, a total of 1517 patients diagnosed with PCa in 6729 males during study period; 3, a total of 5 patients diagnosed with PCa in 504 males during study period;. 4, a total of 7129 patients diagnosed with PCa in 990495 males during study period.

A, self-reported; B, medical records; C, prescription database.

a, current use of statins *vs.* no use of statin; b, current use of statins *vs.* no current use of statins; c, use of statins *vs.* no use of statins; d, any use of statins *vs.* no use of statins; e, ever use of statins *vs.* no use of statins; f, use of statins *vs.* use of anti-hypertensives; g, current use of cholesterol-lowering drugs *vs.* never use of cholesterol-lowering drugs; h, use of pravastatin vs. placebo; I, use of statins *vs.* non-statin lipid-lowering drugs; j, a systematic use of lovastatin *vs.* SEER data; k, use of simvastatin vs. placebo; l, use of fluvastatin vs. placebo; m, use of lovastatin vs. placebo ;

TABLE S2. Characteristics of 13 Case-control Studies Included in the Meta-analysis.

Author, year(country)	Study period(y)	Statin users (N)/ Cases(N)[※]	Statin users (N)/ Controls(N)	Mean follow-up	Description of exposure	Information source	Quality score
Olivan et al.2015(Mediterranean)	6(2006-2011)	152/440	552/1504	NR[$]	a	NR	4
Leung et al. 2013 (China)	8(2000-2008)	26/231	2815/29974	4y	c	C	5
Jespersen et al.2013(Denmark)	13(1997-2010)	7125/41690	35485/208501	NR	a	C	7
Vinogradova et al.2011(U.K.)	10(1998-2008)	2774/14764	11508/61853	28m	c	C	7
Chang et al.2011(Taiwan)	4(2005-2008)	83/ 388	239/1552	NR	e	C	6
Coogan et al.2010(U.S.)	16(1992-2008)	250/1367	178/2007	NR	d	A	3
Agalliu et al.2008(U.S.)	4(2002-2005)	272/1001	244/942	NR	a	A	5
Murtola et al.2007(Finland)	7(1995-2002)	2622/24723	2439/24723	NR	e	C	6
Singal et al.2005(U.S.)	6(1998-2004)	159874/443805[1]		NR	c	C	Unclear[#]
Shannon et al. 2005 (U.S.)	7 (1997-2004)	34/100	99/202	NR	c	C	4
Kaye 2004 (U.K.)	12 (1990–2002)	62/569	204/2488	6.4y	a	C	6
Graaf et al. 2004 (Netherlands)	4 (1995–1998)	NR/186	NR	7.2y	a	C	8
Blais et al. 2000 (Canada)	6 (1988–1994)	NR/78	NR/780	2.7y	n	A	6

[#] Singal et al. is an abstract of ASCO Annual Meeting Proceedings

[※] cases, prostate cancer cases.

$ NR, not reported

1, a total of 26139 were diagnosed with prostate cancer.

A, self-reported; B, medical records; C, prescription database.

a, currrent use of statins vs. no use of statin; b, currrent use of statins vs. no currrent use of statins; c, use of statins vs. no use of statins; d, any use of statins vs. no use of statins; e, ever use of statins vs. no use of statins; f, use of statins vs. use of anti-hypertensives; g, current use of cholesterol-lowering drugs vs. never use of cholesterol-lowering drugs; h, use of pravastatin vs. placebo; I, use of statins vs. non-statin lipid-lowering drugs; j, a systematic use of lovastatin vs. SEER data; k, use of simvastatin vs. placebo; l, use of fluvastatin vs. placebo; m, use of lovastatin vs. placebo ; n, any use of statin vs. use of bile acid-binding resins.

Figure S1. Statins use and risk of localized prostate cancer.

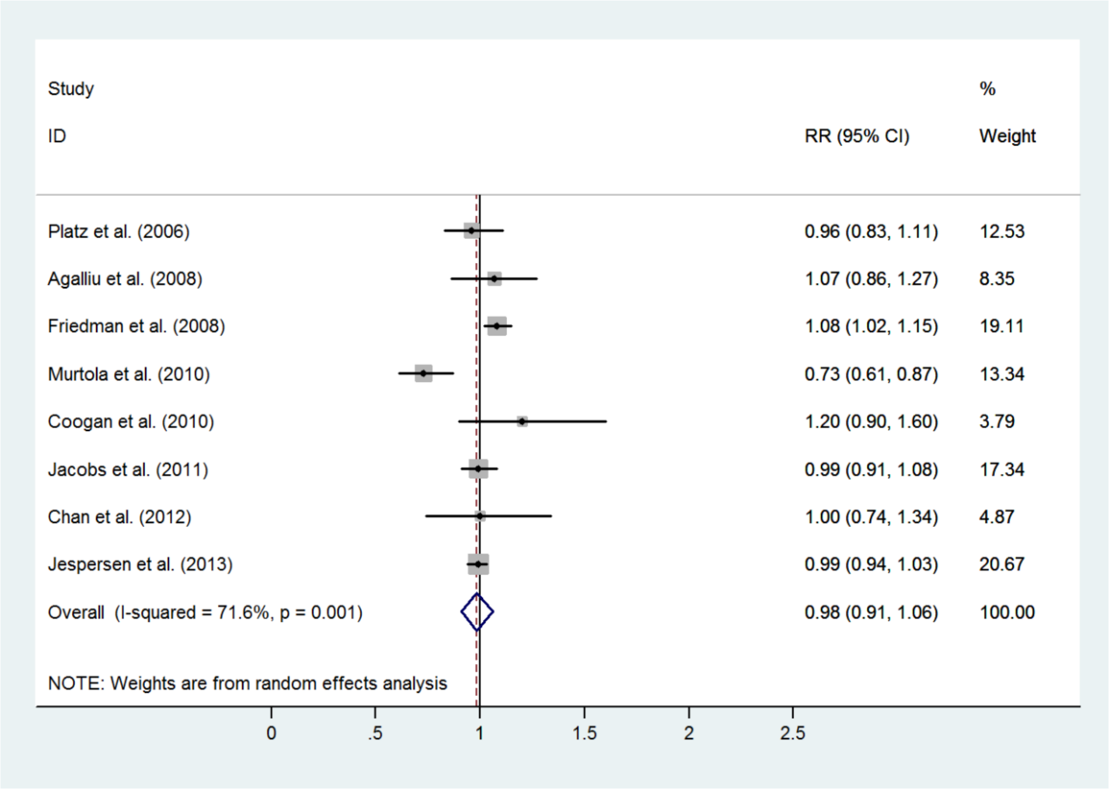

Figure S2. Statins use and risk of high-grade prostate cancer.

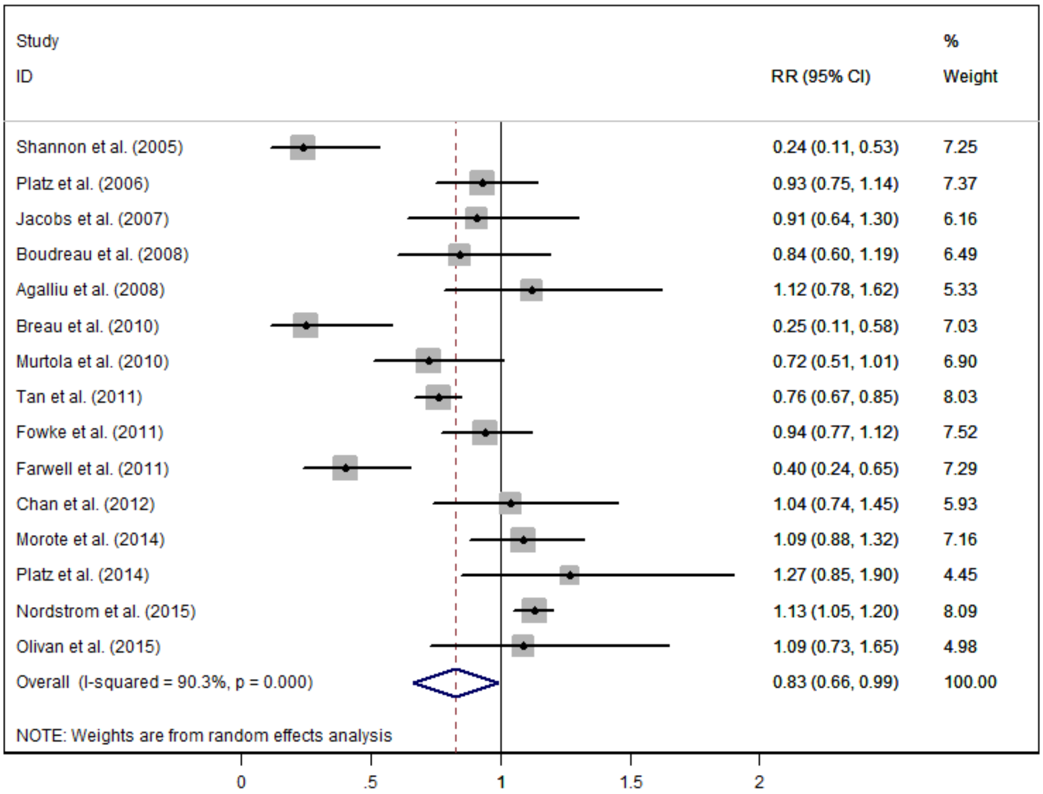

Figure S3. Statins use and risk of low-grade prostate cancer.

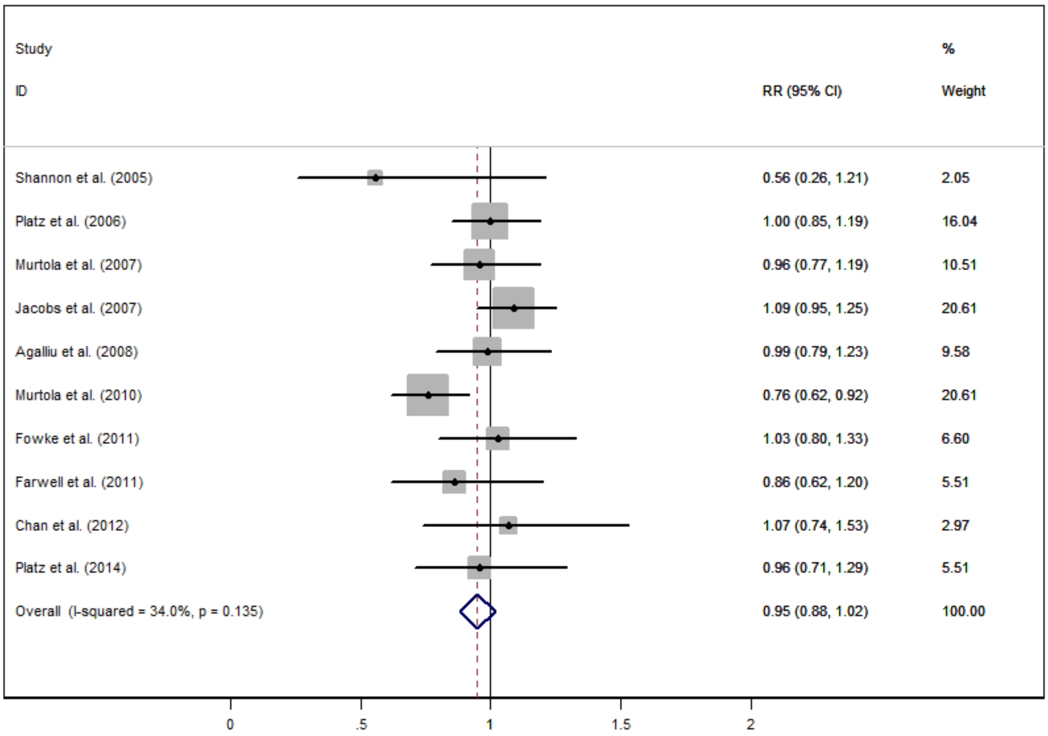

Figure S4. Short-term statins use and risk of total prostate cancer.

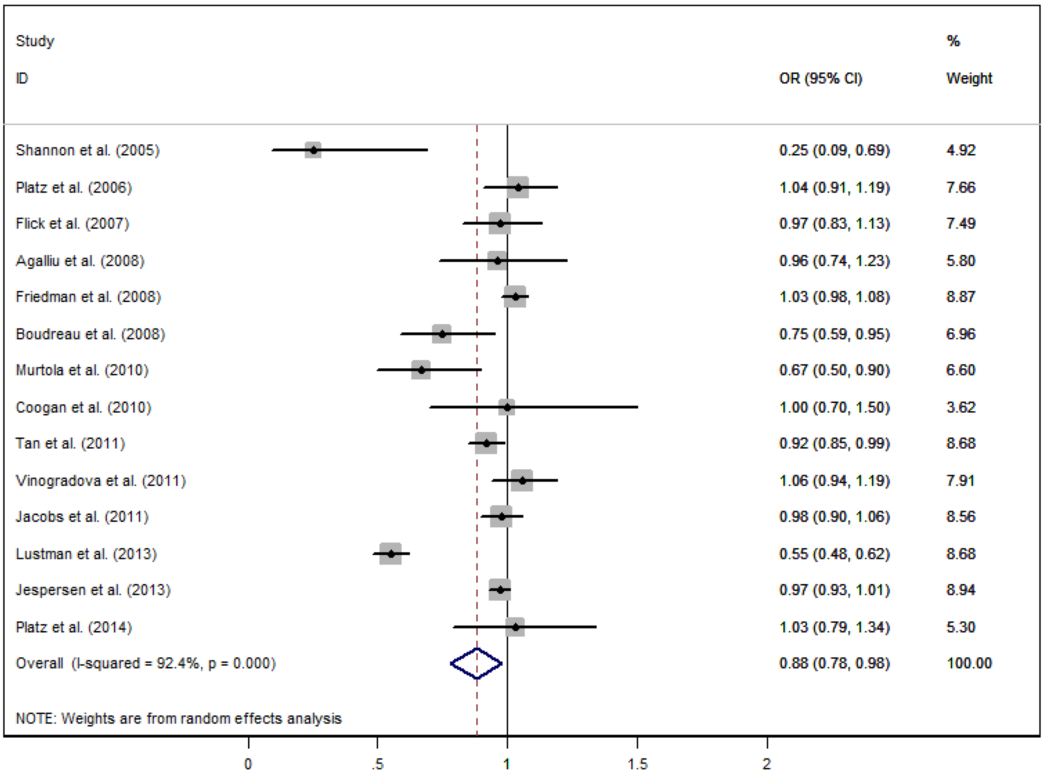

Supplement: Supplementary Information [file srep24521-s1.pdf]
